# Why local air pollution is more than daily peaks: modelling policies in a city in order to avoid premature deaths

**DOI:** 10.1007/s10100-018-0534-y

**Published:** 2018-03-28

**Authors:** Doris A. Behrens, Olivia Koland, Ulrike Leopold-Wildburger

**Affiliations:** 1 School of Mathematics, Cardiff University, Senghennydd Road, Cardiff, CF24 4AG UK; 2 Department of Statistics and Operations Research, University of Graz, Universitaetsstrasse 15/E3, 8010 Graz, Austria

**Keywords:** Predator–prey model, Air pollution, Environmental policy interventions

## Abstract

We use a predator–prey representation of an urban system to analyse how policy interventions can prevent the adverse effects of air pollution on people’s health. *The number of residents* is treated as prey variable, and particulate matter that consists of particles with a diameter of up to 10 micrometres (*PM10*) as predator variable. This representation allows integration of population trends and the effects of environmental interventions on the average level of PM10 concentration (which establishes a baseline for the potential health burden for residents). For the case of Graz, Austria, we illustrate the insights generated regarding the interdependency of market-based and technological pollution controls, and propose an indicator that assesses the cost of delayed interventions by counting additional premature deaths caused by polluted environments.

## Local air pollution in cities

Currently, 92% of the world’s population resides in places where particulate matter, nitrogen dioxide and other local air pollutants exceed recommended limits (UN 2016). More than 8% of the 9 million people who died in 2015 from causes linked to air pollution, are from high income countries (Landrigan et al. 2018); therefore, air pollution cannot be regarded as a “middle- and low-income country problem” or as an issue solely affecting megacities. On the contrary, in Europe’s high-income countries air pollution frequently impacts small urban areas (often reinforced by the topographic and climatic characteristics specific to the region). However, relocating to urban centres is increasingly attractive for people as cities combine the advantages of proximity and diversity—providing jobs, services, swift access to information and innovation—and are more productive than rural areas (e.g. Cervero 2001; UNEP 2011). As a consequence, by 2050, 86% of the population in developed regions are predicted to be living in urban areas compared to 67% in the less developed regions (UN 2011). It should, however, be considered that the positive effects of urban expansion are offset after a certain level due to the proliferation of environmental and health problems that comes with an increase in activities and residents.

The relationship between human population dynamics and environmental conditions is complex and relates to urbanization, migration and land use. In quantifying the impact of population on air pollution, researchers have come to different conclusions depending on which pollutants are under study, in which locations, at what scale, and for which time periods. The variety of impact patterns found (see e.g. De Sherbinin et al. 2007) reflects the multitude of interdependencies observed between different pollutants and regional geographic or climatic conditions (e.g. Neumayer 2002, 2004). Related health issues can be understood as incorporating direct adverse consequences for a person’s physical condition or indirect effects via degrading a person’s level of well-being, making the human body more vulnerable. Research has shifted towards the impact of the latter (for an overview see Welsch 2007, 2009). Variables governing local environmental quality (negatively affecting life satisfaction) are, for example,

- air pollutants e.g. particulate matter,
- nitrogen dioxide or lead (e.g. Rehdanz and Maddison 2005; Welsch 2006; Ferreira et al. 2013; MacKerron and Mourato 2009; Ferreira and Moro 2010) and
- noise (e.g. van Praag and Baarsma 2005; Rehdanz and Maddison 2005).

Each year more than 467,000 premature deaths are caused by air pollution (EEA 2016)—more than 81% of those are caused by high concentrations of particulate matter (Watkiss et al. 2005).^1^ In Europe, particulate matter that consists of particles with a diameter of up to 10 micrometres, denoted by PM10, is the main cause of polluted urban environments (Krzyzanowski 2005; Pey et al. 2009)—and in this paper, we will focus on this type of air pollution due to its relevance in any European context. The non-natural sources for high levels of PM10 concentration include domestic heating, industry including construction, winter road maintenance, and mostly, urban traffic. Motor vehicles emit significant amounts of particulate matter through combustion, brake and tyre wear, and they also contribute to elevated near-road particulate matter concentrations by re-suspending dust present on the road surface (Thorpe and Harrison 2008; Zhu et al. 2002; Kim et al. 2004; Baldauf et al. 2008).

One way of analysing PM10 concentration levels is to study changes of daily indicators of air quality like the number of days exceeding emission limits or daily peaks of nanoparticle concentrations (Kumar et al. 2011).^2^ Our focus lies, however, on understanding the medium- to long-run evolution of urban air quality, i.e. the presence of substantial levels of PM10 to which residents are exposed over time, giving the sustained level of burden on residents on which short-run fluctuations are then superimposed. To integrate the population trend and the effects of environmental interventions on the *average* level of particle concentration we utilise a predator–prey-type model of an urban system. Essentially, this system consists of *residents* and *pollution*: this approach helps to understand how population changes affect the environment, how environmental conditions affect population figures and how different types of environmental policies mediate the relationship between residents and pollution rather than considering environmental change as a mere factor of population size or growth.

At a city level, premature death and quality of life/lifestyle are an important concern for policy makers who seek to maintain urban areas as liveable places. To address these issues, we perceive a predator–prey-type approach as useful for three reasons. (i) It allows visualisation of the dynamic relationship between residents and pollution for urban planners, making the problem more tangible and facilitating dialog. (ii) This approach allows us to go beyond comparative statics, shifting the research focus from analysis of the long-run equilibrium state towards the analysis of the system’s transients. (iii) As a result of (ii) using a dynamic approach allows quantification of damage accumulation over time as a consequence of delayed interventions. To illustrate the usefulness of the predator–prey-type approach to analyse the indicators developed to support urban planners, we have picked a prototypical example of a small urban European centre suffering from air pollution: Graz, Austria. The emphasis lies on the purpose *illustration*. This paper does not intend to be a case study of Graz but should be considered as an invitation to explore the interrelationship of lifestyle, air pollution and premature deaths (via a deterioration of the environment) in a tangible context.

This paper presents a predator–prey model designed to understand the development of (the trend level of) local air quality over time and its dynamic relationship with population size, and its implications for public health modelled by premature deaths. The exercise is illustrated by an example of a prototype European small urban area where we show how behavioural and technical measures can help to mitigate the air pollution problem. To that end, we introduce an indicator that allows the consideration of the negative effect of postponing interventions put in place to save lives.

## A motivating example: the case of Graz, Austria

**Fig. 1:**
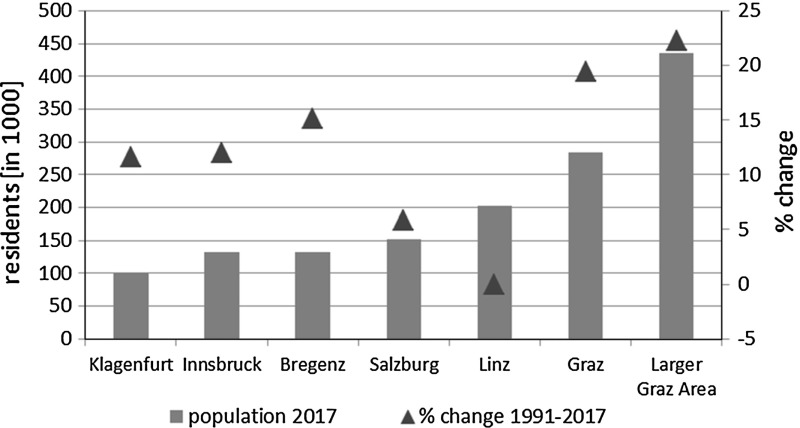
Population levels in Austria’s provincial capitals plus the Larger Graz Area (Graz & GU) 2017 and as %-change of residents between 1991 and 2017. *Source*: Statistics Austria, own illustration

The republic of Austria is a small open economy located in the centre of the European mainland. Most of Austria’s inhabitants (over 8.75M) reside in the catchment area of the country’s capital Vienna (with a reported number of 1,867,582, Statistics Austria, 1.1.2017) or in small urban centres like Graz, Linz or Salzburg.^3^ For more than a decade, Graz and its surroundings have been Austria’s most rapidly growing urban regions (Graz: 283,869 residents; the Larger Graz Area: 434,969 residents, Statistics Austria 1.1.2017). Between 1991 and 2017 the region’s population grew by 22.2% (see Fig. 1). Graz houses little industrial activity but due to its undiminished appeal as a residential area, traffic has substantially expanded. Poor air quality is prevalent—a fact that is dramatically enhanced by the city’s geographical location within a basin at the south-east edge of the Alps causing inversion. As the area thus lacks wind and precipitation, the particle concentration near the ground has to be permanently controlled (but still cannot be sustainably eradicated). The situation is made worse during the winter months.

Over the last decades, the European Union has established an extensive body of environmental legislation and has set standards for various air pollutants. For PM10, a limit of 50 micrograms per cubic metre of air was defined for the daily average value; this limit must not be exceeded on more than 35 days per year. The maximum average level allowed per annum is 40 micrograms PM10 per cubic metre.^4^ In 2005, when the European standard for PM10 was first in effect, 23 of the 27 EU member states reported exceeding PM10 limits. In 2008, the European Commission started to impose penalties for instances of non-compliance with the standards.^5^

Like most of Europe, Austria has struggled to meet the EU limits for air pollution for quite some time. In Graz, the increasing volume of traffic causes 50% of particulate matter emissions (with 39% in winter season), followed by the emissions from industry and commerce (27, 22% in winter) and domestic heating (23, 39% in winter) (Heiden et al. 2008). When considering the major role of raising dust through vehicles, we find that road traffic makes up 62% of total PM10 emissions (Land Steiermark 2003).^6^ To provide the air quality required by EU regulations, local governments have reacted with a range of policies. Austria came up with a national limit on exceedance days even stricter than the EU limit (25 days per year). Despite the implementation of targeted interventions in the city of Graz (based on the ordinance of the government of the federal province of Styria with the first ordinance from 2004, followed by several air quality programmes that have reduced emissions noticeably since 2006), air pollution remains a challenge for public health and the quality of living.

## Modelling premature deaths due to air pollution (high levels of PM10)

In this paper, we seek to quantify the damage caused by shifting the onset of environmental interventions to control poor air quality into the future rather than to instantaneously implement them.^7^ Therefore, it is necessary to understand the intertemporal pattern of population development, the effects of environmental interventions on the evolution of PM10 concentration and the interdependency between population development and PM10 concentrations.^8^ For example, high environmental quality attracts residents (cf. e.g. Gyourko et al. 1999), while poor quality favours relocation to the less-polluted periphery of an urban centre. After a while, when the definite decline in the number of residents (reinforced by control measures) has helped the city centre to recover, potential residents are again attracted and the whole “cycle” starts over again. I.e. the dynamics of the urban system under discussion resemble the structure of a predator–prey model with the number of residents as “prey” and PM10 concentration as “predator”.

While predator–prey systems were originally developed to understand the dynamics of a complex biological system in which two species interact, one as a predator and the other as a prey (see e.g. Lotka 1925; Volterra 1926; Rosenzweig and MacArthur 1963), they have proven to be applicable in many other fields. Applications include economics (Goodwin 1951, 1967), nature conservation in protected areas (Friedl and Behrens 2007), the fall of the Easter Islands (Brander and Taylor 1998), copyright piracy in the music industry (Vaquez and Watt 2011) and urban development (e.g. Orishimo 1987; Camagni 1992; Capello and Faggian 2002; Bednar-Friedl et al. 2016). In the latter context, predator–prey systems have been used to describe the dynamic interaction of residential growth and urban rents (Capello and Faggian 2002), between urban population and per-capita income (Dendrinos and Mullaly 1983), between population and a proxy for the intensity of land use (Orishimo 1987) or between the number of residents and air pollution (Bednar-Friedl et al. 2016).

We follow Bednar-Friedl et al. (2016) and conceptualise residents as the prey variable, since even at zero pollution it would not exhibit more than logistic growth behaviour, and the level of PM10 emission concentration as predator variable. Let us denote the number of residents at year *t* by *R*(*t*) and the level of PM10 concentrations in the air measured in μg/m^3^ averaged over year *t* by *P*(*t*). As addressed above, the rate at which *P*(*t*) changes responds to two quantities: first, the current number of residents *R*(*t*) who contribute to the deterioration (or improvement) of air quality and, second, the per capita rate at which they do so. Let the latter be quantified by the positive parameter $c \ge 0$ that measures how much *PM10 emissions are annually accumulated per resident due to lifestyle and economic activity* like transport or heating. Hence, the urban region’s accumulation rate of PM10 over the course of year *t* can be modelled as *cR*(*t*). If there are more (less) residents, PM10 emissions build up more (less) quickly and are recirculated more (less) intensively in ambient air, ceteris paribus. This implies that the change in the level of PM10 concentrations over the course of year *t* is governed by the expression

1 $$ cR\!\left(t \right)P\!\left(t \right). $$

Denoting the *annual removal rate of PM10 emissions* by $d > 0$, we can quantify the effect of technological environmental interventions. This allows sketching the basic features of changes in the average level of PM10 concentrations as

2 $$ \dot{P}\!\left(t \right) = cR\!\left(t \right)P\!\left(t \right) - dP\!\left(t \right). $$

If urban planners want to maintain urban areas as liveable places with moderate levels of air pollution, they have to actively intervene such that $\dot{P}\!\left(t \right)$ does not become positive. Here, we want to briefly point out the two basic response options: either addressing the exposure to air pollution via behavioural or technical measures. The two parameters epitomising the effects of these two types of control measures we find in Eq. (2):

- Incentivising behaviour change and setting measures to slow down the accumulation (and recirculation) of PM10: These measures aim at reducing parameter *c* in Eq. (2) by e.g. reducing individual traffic or promoting environmentally friendly heating facilities.
- Setting measures to technically remove particles: These measures aim at increasing parameter *d* in Eq. (2) by e.g. street cleaning/rinsing.

Estimating the parameters *c* and *d* for real-world scenarios will be difficult at best. Estimating their magnitude in relation to each other is however sufficient to come up with a better understanding of the urban economic system and how to tackle pollution issues. E.g. if *c* is small in scale while *d* is large, this resembles a city with a “green” lifestyle. If *c* is larger in scale, this describes a city where residents exhibit less environmental awareness and produce a higher level of pollutants per capita. To reach the same level of PM10 in ambient air, then a lot more removal activity is necessary in the latter case, which corresponds to a higher value of parameter *d*. If *d* is small in relation to *c*, the evacuation of pollutants does not happen fast enough and PM10 concentrations build up. In this framework, varying the parameters *c* and *d* can be understood as exploring how the effect of additional interventions (i.e. on top of what is currently done) can change the level of PM10 concentration and its future development.

If we thus use Eq. (2) to describe changes in the average level of PM10 concentrations, in the context of urban systems one element is still missing—and this is the element that distinguishes the model presented here from a prototypical predator–prey system.

We have mentioned above that certain areas are predisposed to higher levels of PM10 concentration due to climatic and/or topographic characteristics which may cause inversion. Then a positive PM10 level, the so-called background emission level, will persist (caused by the economic activity around the region of concern) that does not respond to changes in the current volume of the city’s residents, industry emissions, individual traffic or heating. Let this be embodied in a positive parameter $\tau > 0$, which measures the *average annual level of the background emissions* (that is the constant growth factor of PM10 concentration induced by the economic growth around the city and from increasing levels of commuters between the urban centre and the city’s surroundings). This transforms Eq. (2) into

3 $$ \dot{P}\!\left(t \right) = {{\tau}} + cR\!\left(t \right)P\!\left(t \right) - dP\!\left(t \right). $$

Note that $\tau$ depends on topographic issues. For urban areas located in basins and facing frequent inversions or cities which are located downwind of a heavy polluter, $\tau$ can be expected to be quite high as air cannot be exchanged sufficiently and pollutants stay in the area where they are dispersed by traffic. Hence, Eq. (3) comprises the essential drivers of change in the level of PM10 concentration averaged over the course of year *t*, where change is expressed by a first order, non-autonomous differential equation. To transform Eq. (3) into a predator–prey model we must add the equation for the prey variable that explains the evolution of the number of residents *R*(*t*) for all $t \ge t_{0}$. This, additionally, allows us to directly incorporate the negative health effects of air pollution on human life.

To explain population development over time, we assume that the number of residents faces a *carrying capacity* (denoted by $\varOmega > 0$). In this context, the term “carrying capacity” refers to the number of residents that can be supported by the urban system without reducing the quality of life of its residents (in the long run) or damaging their health (cf. Mathur and Sharma 2016). If cities come close to their carrying capacities, the negative effects of agglomeration outweigh the benefits of urban life. The upper limit for $\varOmega$ depends on external factors such as topography and internal ones such as human behaviour or how public infrastructure is provided. If background emissions are elevated and and/or if the per capita impact of residents to pollution is high because domestic heating is dirty or because people commute a long distance to work, $\varOmega$ is relatively small (compared to what may be considered as “upper bound” from an economic point of view).

Let the parameter $a > 0$ denote the *annual net population growth rate*. Then population growth can be described by a logistic function taking account of the parameters $\varOmega$ and *a*, i.e.

4 $$ aR\!\left(t \right)\left( {1 - \frac{R\!\left(t \right)}{{{\varOmega }}}} \right). $$

The adverse effects of air pollutants on human health provide a rationale for pollution control—the sustained exposure to PM10 pollution leads to respiratory and pulmonary diseases (Brunekreef and Forsberg 2005; Murr and Garza 2009; Andersen et al. 2008), to severe implications for the cardiovascular system (Pope et al. 2004; Dockery and Stone 2007) and to increased morbidity and mortality. The link between residential proximity to major roads and an increased risk to develop adverse health effects is shown in several studies (e.g. Pearson et al. 2000; Wilhelm and Ritz 2003; Finkelstein et al. 2004; Gauderman et al. 2005; McConnell et al. 2006; Samet 2007; Samal et al. 2008). To account for these lethal effects of air pollution on humans we introduce parameter $b > 0$ which represents the *annual dose response relationship between the residents’ mortality and exposure to PM10 pollution*. The percentage of residents that die during year *t* due to exposure to PM10 is determined by *bP*(*T*). Hence, the term *bR*(*T*)*P*(*T*) accounts for the number of premature deaths observed during year *t* due to sustained exposure to PM10. The intertemporal evolution of an urban centre’s size quantified by the number of its residents can therefore be approximated by removing those individuals who experience a premature death, i.e.

5 $$ \dot{R}\!\left(t \right) = aR\!\left(t \right)\left( {1 - \frac{R\!\left(t \right)}{{{\varOmega }}}} \right) - bR\!\left(t \right)P\!\left(t \right). $$

**Fig. 2:**
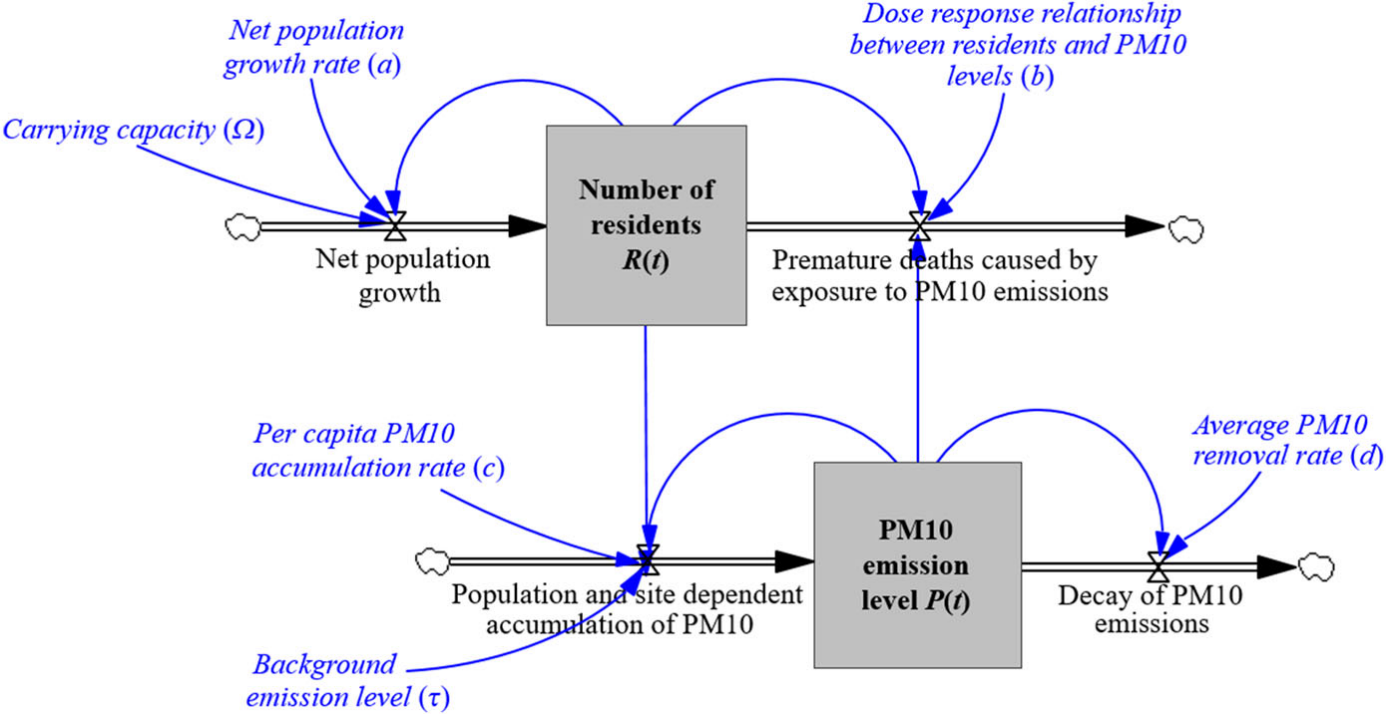
Stocks and flows diagram of the urban (predator–prey) system of mutually dependent population and pollution development (see Eqs. 6a and 6b)

The interrelated dynamics of an urban centre’s level of air pollution and the residents that both cause it and suffer from it are displayed by Fig. 2. This stocks and flows diagram uses block arrows to indicate the flow rates into and out of *P*(*t*) and *R*(*t*). The determining factors of these flow rates are indicated by simple arrows. This allows the nature of the mutual dependency to be visualised: PM10 concentration in ambient air increases more quickly with the number of residents, while the number of residents declines for higher levels of PM10 due to premature deaths. This behaviour can be referred to as that of a “balancing loop” (Sterman 2000), which can be summarised by Eqs. (3) and (5), when adding in initial conditions:

6a $$ \dot{P}\!\left(t \right) = {{\tau}} + \left[ {cR\!\left(t \right) - d} \right]P\!\left(t \right),\quad P\!\left({t_{0} } \right) = P_{0} \ge 0. $$

6b $$ \dot{R}\!\left(t \right) = aR\!\left(t \right)\left( {1 - \frac{R\!\left(t \right)}{{{\varOmega }}}} \right) - bR\!\left(t \right)P\!\left(t \right),\quad R\!\left({t_{0} } \right) = R_{0} > 0. $$

Examining the nature of the relationship between population density and pollution (as displayed by Fig. 2) makes it obvious that missing out on reducing the per capita PM10 accumulation rate (*c*) or on intensifying the annual PM10 removal rate (*d*) implies shorter lifespans for residents. We seek to translate this knowledge into tangible quantities to inform real-world decision-making. A first step towards evaluating the effectiveness of certain environmental policies (embodied in specific values for parameters *c* and *d*) is to determine the number of premature deaths that accumulate over period $\left[ {t_{0} ,T} \right]$. This number can be computed by the following function:

7 $$ \psi \! \left({t_{0} ,T,c,d} \right): = b\mathop \int \limits_{{t_{0} }}^{T} R\!\left(t \right)P\!\left(t \right)dt $$

subject to system (6a) and (6b) for parameters *c* and *d* given. If *c*_0_ and *d*_0_ denote the base case values, respectively, and $\bar{c}$ and $\bar{d}$ the values incorporating one or several interventions, the change in the number of premature deaths due to intervention can be computed by

8 $$ \bar{\psi }\!\left({t_{0} ,T,\bar{c},\bar{d}} \right) := \psi \! \left({t_{0} ,T,c_{0} ,d_{0} } \right) - \psi\! \left({t_{0} ,T,\bar{c},\bar{d}} \right). $$

An urban planner can use $\bar{\psi }\!\left({t_{0} ,T,\bar{c},\bar{d}} \right)$ (see Eq. 8) to determine whether certain environmental policies targeting behavioural changes or indirect and direct controls are regarded as effective enough in preventing premature deaths. In case of ambiguity, delaying decisions is a popular type of behaviour. Therefore, we additionally introduce a second indicator that quantifies the number of premature deaths that are caused by delaying a decision (= doing nothing beyond what is being done already) for *t*_*s*_ years. Therefore, we define

9 $$ \phi\! \left({t_{0} ,t_{s} ,T,\bar{c},\bar{d}} \right): = b\mathop \int \limits_{{t_{0} }}^{{t_{s} }} R\!\left(t \right)P\!\left(t \right)dt + b\mathop \int \limits_{{t_{s} }}^{T} R\!\left(t \right)P\!\left(t \right)dt, $$

subject to system (6a) and (6b), where the first integral in Eq. (9) is computed for the base case parameters values *c*_0_ and *d*_0_ and the second integral for $\bar{c}$ and $\bar{d}$. Then,

10 $$ \bar{\phi}\!\left({t_{0} ,t_{s} ,T,\bar{c},\bar{d}} \right) := \phi \!\left({t_{0} ,t_{s} ,T,\bar{c},\bar{d}} \right) - \psi \!\left({t_{0} ,T,\bar{c},\bar{d}} \right), $$

quantifies the number of premature deaths that can be attributed to delaying the decision to implement measures that change parameter *c*_0_ to $\bar{c}$ and parameter *d*_0_ to $\bar{d}$ by $t_{s} - t_{0}$ years.

In other words, $\bar{\phi }\!\left({t_{0} ,t_{s} ,T,\bar{c},\bar{d}} \right)$ accounts for the lives saved by not waiting $t_{s} - t_{0}$ more years to implement an intervention. We will provide evidence for the informational values of utilising $\bar{\psi }$ and $\bar{\phi }$ for the motivating example of Graz, Austria, below.

## Understanding the issues around handling the urban system and saving lives

Using a model like the one described in the previous section can help to structure and understand a problem and to facilitate dialog. Likewise, the computation of indicators like $\bar{\psi }$ and $\bar{\phi }$ introduced above and defined by Eq. (8) and Eq. (10), respectively, can show benefits of thinking beyond an election cycle when introducing or maintaining interventions and can help to make less myopic decisions. To illustrate this, we calibrate the system (6a, 6b) with empirical data for the city of Graz, Austria.^9^

We use official forecasts of population growth for the Graz municipal area up to 2030 (Magistrat Graz 2012) to derive the parameters *a *= 0.108909 and *Ω* = 295,163. This indicates that if the settlement area of the city of Graz stays within its current limits and the stream of commuters from the surrounding settlement areas remains at its current level, the city has already come quite close to the size that can be supported by the urban system without reducing the health status or the quality of life of its residents. This fact stresses how important it is to sustain the existing environmental interventions and possibly add bespoke measures to keep Graz at a relatively “green” level. Still PM10 is an issue in Graz—and will continue to be due to the city’s location.

If we have a look at the current average PM10 level (27.21 μg/m^3^ in 2016) we find that Graz is well below the PM10 level required by EU regulations (on average 40 μg/m^3^ per year) but still above the level recommended by the World Health Organisation (on average 20 μg/m^3^ per year). To get a crude picture of the disutility associated with exceeding the recommended level of air quality, we seek to “translate” the current PM10 level into premature deaths currently experienced for Graz due to poor air quality. Therefore, we compute the dose response relationship between pollution and residents. The estimation of this value is based on WHO estimates for pre-natural mortality due to higher exposure to PM10 in Austria (Künzli et al. 2000), yielding *b* = 3.7 × 10^−5^ (see Appendix 2).

The level of background emission (that cannot be controlled by the urban planner without collaborating and negotiating with decision makers from surrounding regions with probably different objectives) is estimated based on expert opinion yielding $\tau = 16.25\;{\text{g}}/{\text{m}}^{3}$ (see Appendix 2), while the parameters *c* and *d* cannot be reliably calibrated as independent values. We have to jointly estimate them with a single data series which includes the effect of numerous interventions undertaken since 2004. This leaves us with a high level of uncertainty when selecting suitable combinations of values for *c* and *d*.^10^ Still, understanding the effect of different combinations of *c* and *d* has a lot to contribute to a better understanding of the urban dynamics and how to handle it. This is why we set up three different sets of parameter constellations, {*c*, *d*}, yielding three different scenarios (Scenario 1, 2 and 3) with respect to residential lifestyle and pollution control. All three are of a suitable order of magnitude to serve as framework to represent Graz conditions.

- Scenario 1, $\left\{ {c,  d} \right\} = \left\{ {0.015b,  0.75} \right\}$, (which is included in Table 1) describes a city like Graz with a relatively “green” lifestyle and a substantial level of filtering and road rinsing.

**Table 1:** Base case parameter values calibrated for Graz’s PM10 problem

Parameter	Values	Description
*a*	0.108909	Annual net population growth rate
*Ω*	295,163	Carrying capacity of urban centre
*b*	3.7 × 10^−5^	Annual dose response relationship between the residents’ mortality and exposure to PM10 pollution
$\tau$	16.25	Annual average level of background emissions measured in μg/m^3^
*c*	$b \cdot 0.015$	Annual per capita accumulation of PM10 emissions due to human/economic activity
*d*	0.75	Annual removal rate of PM10 emissions
*R*_0_	280,258	Number of residents at initial time *t *= 0
*P*_0_	27.2	Annual level of PM10 emissions (in μg/m^3^) average over year *t *= 0
*T*	20	planning horizon in years
- Scenario 2, $\left\{ {c,  d} \right\} = \left\{ {0.005b,  0.65} \right\}$, describes a city where the per capita accumulation of PM10 is lower than in scenario 1, i.e. a city with an even “greener” lifestyle but a lower level of *d*-type interventions than observed for the Graz-case.
- Scenario 3, $\left\{ {c,  d} \right\} = \left\{ {0.024b,  0.85} \right\}$, describes a city where the per capita accumulation of PM10 is higher than in scenario 1, i.e. a city with a “less green” lifestyle, while the *d*-type measures are exhaustive (higher than in the Graz-case).

**Fig. 3:**
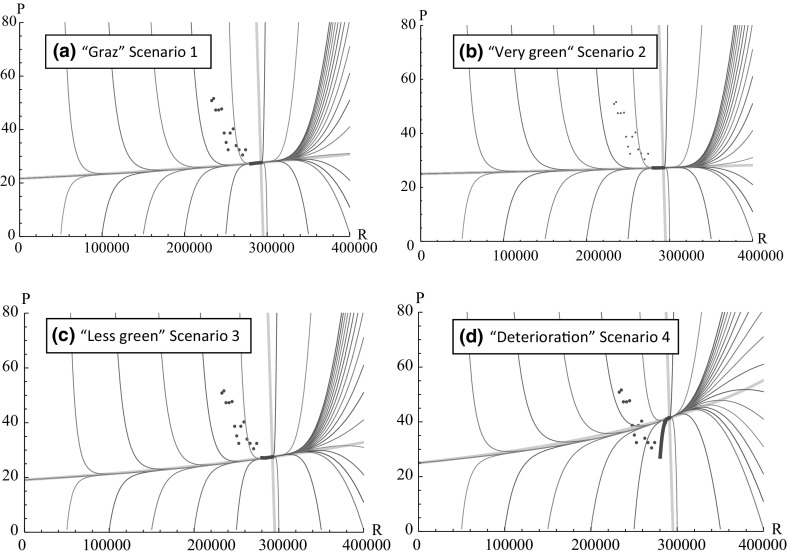
The state space for residents and population **a** for the base case parameter values (top left; see Table 1), **b** the “very green” scenario 2 (top right), **c** the “less green” scenario 3 (bottom left) and **d** the “deterioration” scenario 4 (bottom right); the point cloud represent historical data for the city of Graz since 2002, which follow a downward trend in PM10 concentration (from upper left to lower right)

For the parameter values as described in Table 1 (apart from parameters *c* and *d*), scenarios 1–3 are calibrated such that the urban system approaches roughly the same (feasible) equilibrium^11^ at approximately 292,400 residents and a PM10 level of roughly 27.4 g/m^3^ (see Fig. 3a-c).

Scenario 4 is structurally different from scenarios 1–3 and is created only for one purpose: to show deterioration, i.e. the effect of a “very green” city (like the one described by scenario 2) going “dirty”. This is represented by the parameter values $\left\{ {c,  d} \right\} = \left\{ {0.024b,  0.65} \right\}$ and highlights that not only additional environmental measures are of importance—it is likewise important to maintain a green lifestyle. Let us, for example, do a thought experiment emanating from scenario 2 conditions. These correspond to a very low level of per capita accumulation of PM10 (embodied in parameter *c*). Allowing *c* to increase to the level of scenario 3, ceteris paribus, moves the equilibrium upwards to a PM10 level of roughly 41.5 g/m^3^ (see Fig. 3d). As the effects of interventions tend to fade after a while, the results displayed by Fig. 3d highlight how important it is not to let standards slip.

Figure 3a depicts the urban system’s behaviour for the base case parameter values in the phase plane. The dots represent historical data for the city of Graz from 2002 until 2016, which follow a downward trend in PM10 concentration and an upward trend in the volume of residents. The positively sloped grey line represents the $\dot{P}(t) = 0$ isocline and the negatively sloped grey line represents the $\dot{R}(t) = 0$ isocline. The latter is rather steep as residential numbers hardly respond to changes in pollution, because relocation is a slow process, whereas pollution responds strongly to changes in residents and thus the $\dot{P}(t) = 0$ isocline is rather flat. At the intersection of the isoclines, we find the unique feasible steady state (which is a stable node for the base case parameter set; cf. Bednar-Friedl et al. 2016). The short black trajectory illustrates the evolution of PM10 concentrations from their 2016-level until 2036 (*T *= 20 years). The trajectory is short because the 2016 data point $(R_{0} ,P_{0} )$, recorded in Table 1, is quite close to the equilibrium.

Figures 3a-c show—in the phase plane—different “ways” of progressing from the current population size and pollution to equilibrium conditions. But it is not the equilibrium itself that we are interested in (not even for varying parameter values)—it is the way of getting there. I.e. we are not interested in doing static comparative analyses but in dynamic ones that evaluate the number of premature deaths along the system’s transients.

The current number of premature deaths can be computed by $bR_{0} P_{0}$ utilising the dose response relationship parameter from Table 1 and current population size and pollution, *R*_0_ = 280,258 and $P_{0} = 27.2$ microgram per cubic metre respectively. This tells us that in year $t = 0$ (representing 2016 conditions) an estimated 282 citizens of Graz died due to exposure to PM10 concentrations. Using the base case parameter values (from Table 1) to further calculate numbers $T = 20$ years into the future discloses an increase in emission levels of no more than 1.4%. In year $t = 20$ this will have however caused 5.4% more premature deaths than observed in year $t = 0$ (using the indicator introduced by Eq. 8). The number of premature deaths that will accumulate over the planning horizon of 20 years if no additional environmental measures $(\psi\! \left({0, 20,c,d} \right)$ for parameter *c* and *d* as given by Table 1; cf. Equation 7) are taken is however frightening: Within one generation 5844 citizens of Graz will have had a reduced life span due to poor air quality. Children, the elderly, and those with poor general health are at a high risk (Utell et al. 2005).

To support the transformation of lifestyle and mobility behaviour into a format that is “fit for the future”, cities offer a good starting point. Not only because cities grow continually, but because cities are principally confronted with the problem of offering the different welfare functionalities such as living, income, and environmental quality in a highly effective way. There are different forms in which a city can be organised and around which people organise themselves, and an urban planner can choose between different options to steer the system to meet the goals of providing sufficient access to people, goods/services and clean air. The immediate and future effects of air pollution on individual well-being and general health status indicate why pollution control is of eminent importance for any urban planner. A wide range of measures for clean air are found to address the inner-city transportation system. While behavioural or market-based measures (such as pricing measures) avoid the emission of particles, technical measures (such as filters or street cleaning) reduce the impact of pollutants on air quality or remove particles that are already in the air. The parameters *c* and *d* refer to these two different types of control measures.^12^

If we compare Fig. 3a with Fig. 3b and Fig. 3c we observe that in spite of the fact that all three scenarios are calibrated to yield the same equilibrium level the slopes of the $\dot{P}(t) = 0$ isocline differ. What does that mean? If the lifestyle is very green (scenario 2; Fig. 3b), less filtering is necessary to reach the same equilibrium as in scenario 1. If the lifestyle is less green (scenario 3; Fig. 3c), more filtering is required to reach the same equilibrium as in scenario 1. In other words, what changes is the *effect* of the relationship between residents and pollution. The very green lifestyle scenario, for example, is characterised by a very small *c* value. This indicates that the effect of population growth on the accumulation of pollution is small (therefore the $\dot{P}(t) = 0$ isocline is rather flat). This has an effect on how additional interventions can affect the urban system. Table 2 summarises the effects of 1%-reductions of *c* or 1%-increments of *d* on the number of residents (both interventions to make the city “greener”), PM10 concentration and premature deaths *T* years into the future for $T = 20$. Moreover, Table 2 depicts the effect of the parameter changes above on the number of premature deaths accumulated over the span of a generation (from $t = 0$ until $t = T = 20$).

**Table 2:** Effect of pollution control via behavioural measures (reducing *c*) and technological measures (increasing *d*) on the variables *R*(*T*), *P*(*T*), *bR*(*T*)*P*(*T*), $b\mathop \smallint \nolimits_{0}^{T} R\left(t \right)P\left(t \right)dt$; effects measured for a planning horizon of *T* = 20 years

Variable	Percentage change caused by decreasing c by 1% (%)	Percentage change caused by increasing d by 1% (%)
*Scenario 1*		
Number of residents	0.002	0.010
Level of PM10 emissions in μg/m^3^	0.000	− 1.250
Premature deaths in year t = T = 20	0.000	− 1.305
Premature deaths accumulated over t = T=20 years	− 0.245	− 1.143
*Scenario 2*		
Number of residents	0.001	0.009
Level of PM10 emissions in μg/m^3^	− 0.073	− 1.063
Premature deaths in year t = T = 20	− 0.149	− 1.213
Premature deaths accumulated over t = T=20 years	− 0.081	− 0.984
*Scenario 3*		
Number of residents	0.004	0.012
Level of PM10 emissions in μg/m^3^	− 0.440	− 1.393
Premature deaths in year t =T = 20	− 0.255	− 1.255
Premature deaths accumulated over t = T = 20 years	− 0.389	− 1.285

What Table 2 tells us is that—regardless of the scenario picked—additional *d*-type interventions are more effective than additional *c*-type interventions. This is the case because a successful reduction in pollution (due to reducing *c*) induces additional population growth (due to a decline in *P*), which eliminates part of the beneficial effect of reducing *c*. The effect of *d*-type interventions is however a “straight forward one” as the decay term in the pollution dynamics does not directly interact with the number of (pollution generating) residents. This is an interesting but potentially misleading result as it should not be interpreted as *d*-type interventions being more efficient than changes in the behaviour of the city’s residents—i.e. *generating a bigger bang for a buck*. We are talking about effectiveness here, not cost-efficiency—in fact, at no point in time have we addressed the cost that is associated with a certain effect. What we observe and discuss here are purely the effects of interventions—not the interventions themselves. Moreover, it shall not be forgotten that the relatively low equilibrium level computed for the base case parameters (scenario 1) requires keeping behavioural measures and technical measures as effective as they are today—a key challenge for any growing city.

In Graz, most air-quality measures have become effective in 2006, which has levelled down the equilibrium level and thus lifted to a situation of cleaner air (see historical trajectory in Fig. 3a–d). There is, however, a high level of background emissions (embodied in parameter $\tau$) that is exogenous to Graz’s urban system and uncontrollable by the planner (without collaborating with planners from the surrounding regions and setting up binding agreements on joint interventions). Recall that the parameter $\tau$ is high due to the city’s geographical situation. Our Graz example can be seen as an innovative step to parameterise a predator–prey-type system for a prototype European city. It is the parameters *c* and *d* that can be influenced by a planner. Their reactivity is given by the steepness of the $\dot{P}(t) = 0$ isocline. If the $\dot{P}(t) = 0$ isocline is steeper (which depends on the values estimated for *c* and *d*), then reducing *c* and increasing *d* is much more effective than when the $\dot{P}(t) = 0$ isocline is flatter. If behavioural or technical measures do not matter (any more), the equilibrium level of PM10 exclusively depends on $\tau$.

One issue has been left unaddressed so far. Usually, implementing interventions is not an all or nothing exercise—often the question is not whether at all to implement a certain intervention. Rather there is an inclination to shift decisions into the future (often into the next election cycle). Then the question to be answered is what is lost in terms of human lives if we do so. In other words, an urban planner has to trade off premature death and expenditures for additional measures and needs to be well-informed about the consequences of his or her decisions. To illustrate this point, assume that we could travel back in time by four years, which actually was the instant when we (the authors of this paper) first discussed the behavioural approach of pollution outlined here, and ask ourselves what we have missed by not intervening earlier. Using Eq. (10) and the base case parameters summarised in Table 1 the results would be the following:

Starting with 2013 conditions, reducing *c* by 1%, would have saved almost 57 additional lives *by not delaying the decision until today* and reducing *c* by 1% now (= 4 years later). Increasing the parameter *d* by 1% in a timely manner would have been even more effective. Not delaying the decision to implement *d*-type measures (that increase the parameter by 1%) would have saved 64 lives (compared to implementing the identical intervention four years later). The same exercise can be carried out in a forward looking way—using Eqs. (6a, 6b) to model the future development of the urban problem. Any “saving” in intervention cost by simply shifting its onset into the future can therefore be confronted with the human lives lost due to postponing the intervention. As mentioned above, this allows urban planners to make (more) informed decisions when it comes to combining environmental measures that affect an urban system in structurally different ways.

## Conclusions

A city is a human–environment system embedded in an economic structure. Urban air pollution is often used as an indicator for the quality of life in cities with a direct effect on public health. However, air pollution is not independent from quality of life and public health. A deterioration of local air quality can be caused by an increasing population density, which is enhanced due to locational welfare factors such as the supply of goods and services, green environment, and accessibility. In other words, air pollution is not necessarily an independent *measure* for the quality of life in a particular area but rather a *variable* in an urban system. Therefore, in this paper we have integrated the evolution of city size and the response of air quality with respect to the residents’ growth in a predator–prey representation of an urban system. The modelling exercise provides insight into the complexity that the handling of an urban pollution problem poses to a planner in the longer run (cf. Bednar-Friedl et al. 2016). The predator–prey model presented here not only allows to develop a better understanding of the underlying structure of the intertemporal development of local air quality and its interlinkage with residential dynamics—it also shows how to capture and understand the number and evolution of premature deaths caused by poor air quality in a small urban centre. Moreover, the model enables a planner to develop a refined understanding of the relative effectiveness of environmental measures (while not of their cost-efficiency), which is most helpful in terms of policy intervention. In this context, the effectiveness of interventions is dependent on how green the lifestyle of the urban residents is and how ambitiously air quality measures have been set by policy makers.

We have parameterised the proposed model for a prototypical urban centre: Graz, Austria. For this illustrative example, we have pursued a scenario analysis. The analysis shows that setting additional measures to support the evacuation and dilution of pollutants after pollution has built up is more effective than measures which incentivise behaviour change to preventatively slow down the rise in PM10 concentration. This by no means should be understood as a recommendation that Graz should “forget” behavioural measures in the future. By contrast, the challenge and the crucial point is to sustain the current effectiveness of behavioural measures. Furthermore, the current PM10 level (already very close to its equilibrium level) can only be sustained, if Graz continues with today’s portfolio of air quality measures or a comparable bundle of policies in terms of their effectiveness.

While it is well known what kind of measures need to be installed to improve the situation in inner-city areas (see e.g. Bednar-Friedl et al. 2011), it is less known how severely public health can be affected by a delay of regulating activities. Even for a city like Graz with a situation that is already quite “green” in terms of living quality, the number of premature deaths due to poor air quality is substantial. Under current—favourable—conditions still an estimated 5700 lives will end prematurely within the next 20 years due to poor air quality. Similarly substantial is the number of premature deaths tolerated by shifting the onset of interventions into the future. The model presented in this paper and the indicators developed based on it allow better understanding of the urban problem and quantify the adverse consequences of not making decisions. This facilitates dialog as a basis of improvement—and this is essential for cities like Graz, which are attractive to residents and commuters from a large catchment area, located in a basin prone to inversion. Here, urban planners will be forced to collaborate with decision makers of neighbouring regions. Individual traffic and heating will be core issues for environmental policy not only for the inner-city regions but also for the urban hinterland. Otherwise, the increase in background emission is inevitable and its effect will be reinforced by individual traffic, reducing the quality of life in the inner city. This in turn favours relocation of the urban population to the surrounding areas, which increases the volume of commuters and PM10 background emissions. To escape this vicious cycle while keeping residents engaged in a “green” lifestyle is certainly a demanding task for any urban planner—now and in the future.

## Appendices

### Appendix 1: The urban system’s equilibrium

The predator–prey type system (Eqs. 6a, 6b) has three steady states, determined by the intersection of the isoclines, i.e.

$$ \hat{R}_{1} = 0,\;\hat{P}_{1} = \frac{\tau }{d};\quad \hat{R}_{2,3} = \frac{a(d + c\varOmega ) \pm A}{2ac},\hat{P}_{2,3} = - \frac{a (d - c\varOmega ) \pm A}{2bc\varOmega }, $$

where $A: = \sqrt {a\,(a\,(d - c\varOmega )^{2} + 4bc\varOmega \tau )}$. The steady state $E_{1} = (\hat{R}_{{{\kern 1pt} 1}} ,\;\hat{P}_{1} )$ is characterised by zero residents but a strictly positive level of PM10 emissions as long as the level of background emissions $\tau$ is positive. Moreover $E_{1}$ is inversely related to the control-induced decay rate *d*. Inspection of $\hat{R}_{2}$ reveals that $\hat{R}_{2} > \varOmega$. Therefore, $A > a(d - c\varOmega )$ which implies that $\hat{P}_{2} < 0$. Hence, $E_{2} = (\hat{R}_{{{\kern 1pt} 2}} ,\;\hat{P}_{2} )$ is not feasible as a negative emission level has to be excluded. Finally, when both the numerator in $\hat{R}_{3}$ and $(d - c\varOmega )$ are positive (which is ensured by a sufficiently large value of *d*), an equilibrium solution exists with $\hat{R}_{3} \in (0,\varOmega )$ and $\hat{P}_{3} > 0$.

### Appendix 2: Assigning parameters to the model

We assume that the future evolution of the number of Graz’s residents will roughly follow level and pace of the population prognosis provided by Magistrat Graz (2012) up until 2030, ceteris paribus. The net growth rate *a* of the continuous-time population dynamics needs to be jointly estimated with the city’s carrying capacity $\varOmega$. To do so, we utilise the discrete-time analogue of the population dynamics given by Eq. (6b) but neglect the negative feedback effect of pollution on the number of residents, i.e.,

11 $$ R_{i + 1} - R_{i} = \alpha R_{i} \left( {1 - \frac{{R_{i} }}{\varOmega }} \right)\quad \Leftrightarrow \quad \frac{{R_{i + 1} - R_{i} }}{{R_{i} }} = \alpha \left( {1 - \frac{{R_{i} }}{\varOmega }} \right). $$

By means of Eq. (11) the estimation of $\alpha$ and $\varOmega$ relies on the observed population data provided for year $i = 2011$ and the anticipated population data for year $i = 2030$ (Magistrat Graz 2012). Simultaneously solving the corresponding equations,

12 $$ 3,\!428/261,\!540 - \alpha \left( {1 - 261,\!540/\varOmega } \right) = 0. $$

13 $$ 739/288,\!594 - \alpha \left( {1 - 288,\!594/{{\varOmega }}} \right) = 0. $$

yields $\alpha = 0.115061$ and *Ω* = 295,163. Then, *a* is equal to the continuous-time equivalent of the estimated growth rate $\alpha$, i.e., $a = ln\left( {1 + \alpha } \right) = ln\left( {1.115061} \right) = 0.108909$, while the estimate for the carrying capacity of the continuous-time model remains *Ω* = 295,163 (since it is not a flow rate).

From a WHO study (Künzli et al. 2000) we are informed about the fact that, in a population of one million residents, particulate matter (PM) emissions of 10 μg/m^3^ cause 370 additional deaths per annum. This implies $b \approx 0.000037$ (here we abstain from introducing a different notation for the flow rates of the continuous and the discrete version of the population dynamics as the calibrated values of parameter *b* nearly coincide due to the parameter’s small magnitude); a lower bound on the dose–response parameter value is given by $b_{inf} = 0.000023$, an upper bound by $b_{sup} = 0.000052$.

According to regional experts (C. Kurz, D. Öttl, personal communication 2015)^13^ Graz’s PM10 background emissions from external sources make up about 50% of the currently prevailing PM concentration. I.e., without human/economic activities, the PM concentration in Graz would rather quickly drop by 50% (in terms of the yearly average).^14^ Most recent data from Graz Don Bosco (from 2013) report that the annual level of PM emissions amounts 32.5 μg/m^3^ on average.^15^ We thus estimate the parameter $\tau$ in Eq. (6a) as being equal to 16.25 μg/m^3^ (in terms of an annual average).

## Footnotes

1 A number of studies have focused on the health effects arising from ambient particulate matter (PM) concentrations for population health. For example, Georgopoulos et al. (2005) determined population exposure by matching people's activity patterns using Geographical Information System mapping. More recently, Bravo et al. (2012) compared exposure estimation methods for air pollutants, i.e. ambient monitoring data and regional air quality simulation. Soares et al. (2014) presented a population exposure model for urban settings with application to the city of Helsinki. Lee et al. (2015) developed a method to quantify the effects on global premature mortality of changes to PM precursor emissions. Miranda et al. (2016) applied integrated assessment methodologies for determining the effect of emission reductions to population exposure in urban areas. Aleksandropoulou and Lazaridis (2017) assessed population exposure based on combining population maps with maps of the PM concentrations.

2 The changes in daily PM10 indicators usually depend on characteristics like weekday, season, heating, wind, precipitation, temperature or fog.

3 Graz (283,869 residents), Linz (203,012) and Salzburg (152,367) are Austria’s second, third and fourth largest cities (Statistics Austria, 1.1.2017).

4 Note that the WHO Air Quality Guidelines recommend that the annual mean of PM10 must not exceed 20 micrograms per cubic metre (WHO 2006)—as opposed to the 40 micrograms per cubic metre that are mandatory within the European Union.

5 The EU’s Clean Air Directives include: Air Quality Framework Directive 96/62/EC; first daughter directive 1999/30/EC; second daughter directive, Council Directive on Ambient Air Quality and Cleaner Air for Europe 2008/50/EC. Reference for directive 1999/30/EC: European Community, 1999. Council Directive 1999/39/EC of 22 April 1999 regulating to limit values for sulphur dioxide, nitrogen dioxide and oxides of nitrogen, particulate matter and lead in ambient air. Official Journal of the European Communities L163, 0041–0060.

6 PM10 is a well-documented and extremely harmful pollutant in the Graz area and has thus been measured by the local monitoring network since 2001.

7 Note that here we seek to understand the relative effectiveness of interventions and the related behaviour of an urban system over time. Addressing recommended or even optimal levels of interventions goes beyond the scope of the present paper.

8 Since four out of five premature deaths caused by poor air quality are due to high concentrations of particulate matter (Watkiss et al. 2005), in this paper we focus on PM10 concentrations.

9 A description of the calibration procedure for the population dynamics is provided in Appendix 2.

10 Due to the uncertainty involved, we stress here that the parameter values chosen for *c* and *d* serve illustrative purposes only and should not be used to predict the development of the air quality in Graz.

11 The feasible equilibrium is displayed in Appendix 1.

12 From a strategic point of view, command-and-control measures are easier to implement and less expensive to enforce (Levinson and Shetty 1992; Davis 2008) if compared to market-based instruments like pricing individual transport or subsidizing the restoration of private dwellings. Command-and-control measures include standards or regulations such as the retrofitting of motor vehicle particle systems with filters or energy/emission standards for domestic heating systems. Moreover, “soft” behavioral measures such as air quality alerts are less expensive and less politically controversial (cf. e.g. Tribby et al. 2013; Zivin and Neidell 2009) than “hard” ones such as congestion pricing, while the effectiveness of soft measures on individual driving is limited (Bamberg et al. 2011; Bannister 2007).
